# Turn up the red: MADS-RIN-DIVARICATA1 module positively regulates carotenoid biosynthesis in nonclimacteric pepper fruits

**DOI:** 10.1093/plphys/kiaf104

**Published:** 2025-03-19

**Authors:** Lara Pereira, Chong Teng

**Affiliations:** Assistant Features Editor, Plant Physiology, American Society of Plant Biologists; Ecology and Evolutionary Biology, School of Biosciences, University of Sheffield, Sheffield S10 2TN, UK; Assistant Features Editor, Plant Physiology, American Society of Plant Biologists; Department of Plant Sciences and Genome Center, University of California, Davis, CA 95616, USA

Peppers are used worldwide for their nutritional value, unique taste, and vibrant colors. Unripe pepper fruits display shades such as white, purple, or green, transitioning to carotenoid-based colors—yellow, orange, and red—as they ripen. Carotenoids, lipid-soluble molecules essential for photosynthesis and photoprotection, are synthesized through well-characterized enzymes (Rodriguez-Uribe et al. 2012; Gómez-García and Ochoa-Alejo 2013), including phytoene synthase 1 (PSY1) and capsanthin/capsorubin synthase (CCS) (Fig.). The transcriptional network promoting carotenoid synthesis, among other traits, during ripening is partially elucidated in tomato, a model species for fruit ripening. However, as a nonclimacteric fruit, pepper relies on potentially different and less-understood regulatory mechanisms for carotenoid biosynthesis. Recently, the MYELOBLASTOSIS (MYB) containing two repeats (R2R3-MYB) transcription factor DIVARICATA1 was shown to be a positive regulator of capsanthin content through direct activation of *PSY1* and *CCS* transcription (Song et al. 2023). Interestingly, the well-studied ripening regulator, MADS box transcription factor RIPENING INHIBITOR (MADS-RIN), key in triggering climacteric ripening, is coexpressed with DIVARICATA1 and activates its promoter in pepper. These findings open the question of whether these 2 transcription factors interact to modulate carotenoid biosynthesis (Song et al. 2023).

**Figure.**
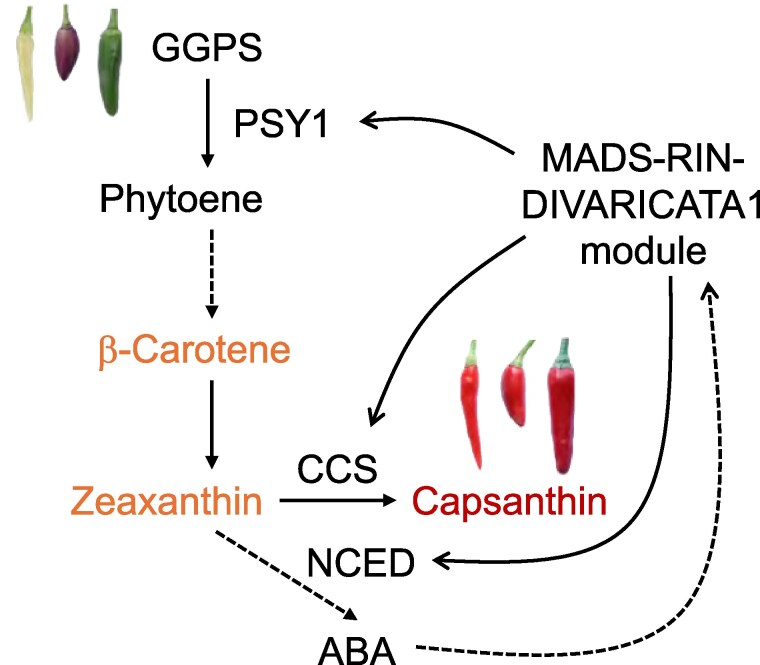
A schematic overview of the MADS-RIN-DIVARICATA1 module positively regulating carotenoid biosynthesis in nonclimacteric pepper fruits. This module activates the expression of *PSY1* and *CCS* genes, converting lipid precursors of carotenoids in unripe fruits into yellow, orange, and red pigments, including capsanthin. Additionally, MADS-RIN enhances ABA synthesis and collaborates with DIVARICATA1 to further promote carotenoid biosynthesis. Figure is modified from Song et al. (2023) and Wang et al. (2025).

In a recent article published in *Plant Physiology*, Wang et al. (2025) identified a coexpression module containing both *MADS-RIN* and *DIVARICATA1* through a weighted gene coexpression network analysis, a powerful computational method for elucidating gene–gene relationships using expression data. Transcript levels of *MADS-RIN* and *DIVARICATA1* strongly correlated with the enzymes *PSY1*, *CCS*, and *β-CAROTENE HYDROXYLASE* (*CHYB1*), involved in the capsanthin biosynthetic pathway, and with *9-CIS-EPOXYCAROTENOID DIOXYGENASE 3* (*NCED3*) and *ABSCISIC ACID*  *8'-HYDROXYLASE* (*ABA8ox*), part of the biosynthetic pathway of the plant hormone abscisic acid (ABA). Maximum gene expression also correlated with capsanthin levels. The authors used virus-induced gene silencing to evaluate the impact of *MADS-RIN* on carotenoid synthesis and observed a strong reduction of *PSY1*, *CCS*, *CHYB1*, and *DIVARICATA1* transcript levels as well as of capsanthin content. Interestingly, MADS-RIN can directly bind to specific CArG-box elements located in the promoters of *PSY1*, *CCS*, and *DIVARICATA1* in vivo and in vitro, suggesting that MADS-RIN is the direct regulator of these genes. Furthermore, MADS-RIN and DIVARICATA1 proteins interacted, forming a regulatory complex that synergistically boosted *PSY1* and *CCS* expression (Fig.), highlighting their cooperative role in carotenoid biosynthesis.

Pepper is classically considered a nonclimacteric species in contrast with the model species tomato, even though both are both members of the Solanaceae family. Traditionally, climacteric fruits such as tomato rely on ethylene to ripen properly, while in nonclimacteric fruits, ethylene seems to have a minor role in ripening that is instead triggered by ABA (Cherian et al. 2014). ABA seems to act upstream of *DIVARICATA1*; however, this transcription factor clearly mediates capsanthin biosynthesis (Song et al. 2023). In addition, since ABA is a downstream product of the carotenoid biosynthetic pathway, a feedback loop might play a role in this process. Wang et al. (2025) extended this knowledge by showing that genes involved in ABA biosynthetic pathway were upregulated in the *MADS-RIN-DIVARICATA1* coexpression module, and silencing *MADS-RIN* reduced ABA synthase expression. Moreover, MADS-RIN directly bound ABA synthase gene promoters, implicating *MADS-RIN* as a central regulator initiating ABA production to drive pepper fruit ripening.

MADS-RIN, as well as other regulators such as the NAC transcription factor NONRIPENING (NAC-NOR), promote fruit ripening not only in climacteric but also in nonclimacteric fruits. suggesting that the transcriptional network is conserved, at least to some extent (Martín-Pizarro et al. 2021; Zenoni et al. 2023). MADS-RIN affects ripening not only in an ethylene-dependent but also in an ethylene-independent way; in fact, it likely plays a role in fruit ripening upstream of ethylene (Li et al. 2022). In addition, when the tomato *rin* mutant overexpressed a wild-type allele of the pepper *MADS-RIN* gene, ripening was restored, including carotenoid biosynthesis (Dong et al. 2014). Therefore, the key role of this transcription factor in carotenoid biosynthesis in pepper is not surprising. An interesting question to be explored in follow-up research would be whether other ripening-related processes, such as flesh softening, volatile biosynthesis, and cell wall modifications, are also triggered by MADS-RIN in a fruit classified as nonclimacteric, such as pepper.

MADS transcription factors, including MADS-RIN, usually form protein complexes with partners defining their molecular function. In tomato, these regulatory complexes bind to the promoters of ripening-responsive genes, promoting ethylene autocatalytic synthesis as well as the physiological changes driven by this hormone (Zhang et al. 2018). Curiously, R2R3-MYB transcription factors have been predominantly shown to regulate flavonoid/anthocyanin biosynthesis during fruit ripening, rather than carotenoid biosynthesis, through MYB–bHLH–WD40 complexes (Jaakola 2013). Wang et al. (2025) demonstrated here that in nonclimacteric pepper, MADS-RIN physically interacts with the R2R3-MYB DIVARICATA1, unraveling a novel mechanism contributing to carotenoid biosynthesis. Given the major role of MADS-RIN in triggering fruit ripening, it is possible that other ripening-related processes could also be regulated by this complex.

The research presented in this article highlights the pivotal role of the MADS-RIN-DIVARICATA1 module in regulating carotenoid biosynthesis in nonclimacteric pepper fruits. Further insights into the interplay between *MADS-RIN*, *DIVARICATA1*, and potentially other transcription factors, as well as their impact in ABA biosynthesis, would reveal novel approaches to optimize fruit quality, fruit color, and shelf life in pepper and other nonclimacteric fruits.

## Data Availability

No new data were generated or analyzed in support of this research.
